# Replica exchange molecular dynamics for Li-intercalation in graphite: a new solution for an old problem†

**DOI:** 10.1039/d3sc06107h

**Published:** 2024-01-16

**Authors:** Heesoo Park, David S. Wragg, Alexey Y. Koposov

**Affiliations:** a Centre for Material Science and Nanotechnology, Department of Chemistry, University of Oslo P.O. Box 1033, Blindern Oslo 0371 Norway heesoo.park@smn.uio.no alexey.koposov@kjemi.uio.no; b Department of Battery Technology, Institute for Energy Technology (IFE) Instituttveien 18, Kjeller 2027 Norway

## Abstract

Li intercalation and graphite stacking have been extensively studied because of the importance of graphite in commercial Li-ion batteries. Despite this attention, there are still questions about the atomistic structures of the intermediate states that exist during lithiation, especially when phase dynamics cause a disordered Li distribution. The Li migration event (diffusion coefficient of 10^−5^ nm^2^ ns^−1^) makes it difficult to explore the various Li-intercalation configurations in conventional molecular dynamics (MD) simulations with an affordable simulation timescale. To overcome these limitations, we conducted a comprehensive study using replica-exchange molecular dynamics (REMD) in combination with the ReaxFF force field. This approach allowed us to study the behavior of Li-intercalated graphite from any starting arrangement of Li at any value of *x* in Li_*x*_C_6_. Our focus was on analyzing the energetic favorability differences between the relaxed structures. We rationalized the trends in formation energy on the basis of observed structural features, identifying three main structural features that cooperatively control Li rearrangement in graphite: Li distribution, graphite stacking mode and gallery height (graphene layer spacing). We also observed a tendency for clustering of Li, which could lead to dynamic local structures that approximate the staging models used to explain intercalation into graphite.

## Abstract 2

Li intercalation and graphite stacking have been extensively studied because of the importance of graphite in commercial Li-ion batteries.

## Graphical abstract

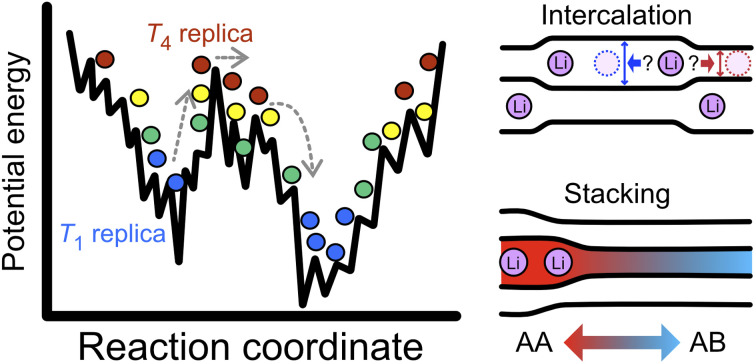

## Introduction

In the past decade, extensive studies have been carried out to understand the correlations between structure and reversibility of active electrode materials,^1–4^ which control long-term cycling stability in modern alkali-metal ion batteries.^5^ Active materials in batteries undergo dynamic structural and morphological transformations during electrochemical cycling. Generally, materials with the intercalation mechanism are the most stable under cycling.^1,6,7^ However, irreversible structural transformations, which accumulate during the operation of a battery, result in long-term degradation of active materials and, thus, capacity fading. Knowledge of the chemical operation mechanisms of active materials is required to understand the chemistry of the degradation processes and engineer chemical pathways to improve material stability. Typically, the summary of the operating mechanisms involves descriptions of phases formed during cycling and the sequence of transitions from one phase to another. To understand the phase evolution, most structural studies (even studies of local structure) adopt homogeneous models, which treat the material as a periodic repetition of the same structural motif. However, a battery under electrochemical cycling is an extremely dynamic chemical system, and therefore, homogeneous models can not accurately describe the structures of the active materials under non-equilibrium conditions during (dis)charge processes. Several experimental studies have demonstrated inhomogeneous lithium (Li) distribution in electrodes over μm to nm length scales, suggesting that similar effects could be expected at the atomic scale.^8–10^

Graphite is the state-of-the-art anode material for modern Li-ion batteries (LIBs) with stable electrochemical performance and reasonable capacity, however, its operating mechanism is still debated in the literature. It has a layered structure made up of stacks of graphene sheets separated by spaces, which we will refer to as “galleries”. This structure allows graphite to host guest ions and molecules within the galleries *via* an intercalation mechanism. This process was described in an early crystallographic study by Rüdorff and Hofmann.^11^ This work introduced the concept of “staged” intercalation in graphite (referred to herein as the R–H model), which has become the cornerstone for most descriptions of the cycling mechanism of graphite in LIBs. In the R–H model, the guest species are intercalated in every *n*-th gallery (*n* then becomes the stage number, *i.e.* one lithiated gallery for every three layers = stage 3), creating an ordered array of fully occupied and completely empty galleries, which explains the change in the crystallographic *c*-axis lattice spacings (*i.e.*, in the direction perpendicular to the graphene plane, the *z*-axis of the REMD simulation box) measured at different levels of intercalation. The graphene sheets in this model are completely flat and inflexible. Missyul *et al.*^12^ have classified the crystal structures for lithiated graphite according to the R–H model, on the basis of powder X-ray diffraction (XRD) data, and a dilute Li-intercalated graphite (LIG) structure reported by Dahn was also rationalized on this basis.^13^ Theoretical research at the atomic level confirmed the energetic stability of the staging structures.^14,15^ In 1969, Daumas and Hérold^16^ proposed an alternative model (D–H model) where the intercalating species tend to cluster together and form a periodic arrangement of islands. This model also suggests that the graphene layers deform (or bend) around the clustered intercalants. Recent studies employing *operando* X-ray diffraction have elucidated the phase transitions in lithiated graphite and explained them on the basis of the R–H and D–H stage models.^17–21^

Furthermore, the R–H and D–H models can only account for “dilute” distributions of Li atoms using partial occupancy of the Li sites. Although electron microscopy experiments on lithiated graphite provide experimental evidence for gallery height variation,^22–24^ and some of these show evidence of Li clustering with a degree of order that resembles the D–H model,^25–27^ it has not as yet been included in the models used to fit the X-ray and neutron diffraction data. In theoretical studies, the cost of compute time has limited density functional theory (DFT) calculations, so flat graphene layers were adopted by most models.^14,15^ Conventional molecular dynamics (MD) simulations are ineffective because the rate of the Li migration event—diffusion coefficient^28^ of 10^−5^ nm^2^ ns^−1^ (=10^−10^ cm^2^ s^−1^)—is slow compared to the timescale of the simulations (which normally only cover up to a few hundreds of nanoseconds).

We deployed replica-exchange molecular dynamics (REMD) simulations to explain the relative energetical favorability of varying Li intercalations in graphite and provide detailed atomistic structures for a range of possible Li arrangements at different Li concentrations, including inhomogeneous Li-intercalation. REMD has been used extensively to understand the behavior of molecules and atoms in complex systems such as peptide self-assembly and protein folding. Due to the charge-transfer nature of Li, a ReaxFF reactive force field was used in the simulations to account for the effect of Li concentration on the local structure energetics of Li adsorption and migration on graphite. In this work, this method was used to accelerate Li migration and allowed us to obtain representative structures in a reasonable timescale. We investigated the structures of Li_*x*_C_6_ systems starting from various homogeneous and inhomogeneous Li distributions for all values of *x* and compared the structural configurations by correlating the Li concentration in graphite with the stacking mode and local Li distribution. The three main features that control structural stability are the Li distribution (especially clustering), graphite stacking mode, and gallery height, which are dynamic and dependent on one another.

## Inhomogeneous Li intercalation graphite modeling

**Fig. 1:**
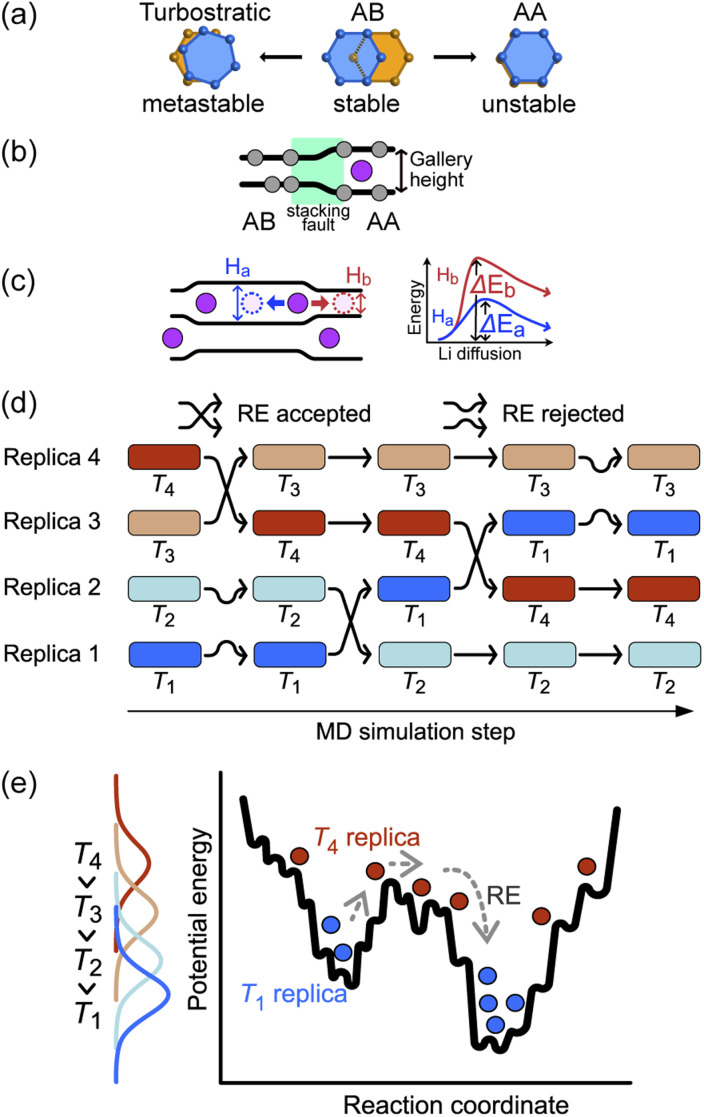
(a) Graphite stacking modes. The orange and blue rings belong to adjacent graphene layers in the stacked structure (b) variations in the height of the graphite gallery between vacant and occupied Li sites, leading to a stacking fault (green region). (c) Gallery height variations with multiple Li intercalations. The Li atom diffuses toward the vacant site (following the blue arrow) with increased gallery height *H*_a_ due to a neighboring Li atom, rather than following the red arrow into the area with standard graphitic spacing *H*_b_ (d) schematic of REMD. Several simultaneous MD simulations at different temperatures are completed between exchange attempts, while the exchange acceptance is decided on the basis of Metropolis criteria. (e) A schematic potential energy landscape and histograms of the trajectories at an individual temperature.

The basic stacking unit of crystalline graphite consists of two graphene layers with a spacing of 3.35 Å between them; the layers are offset so that one of the atoms from the layer above is positioned on the top six-membered ring center of the lower graphene sheet (Fig. 1a). This motif is repeated to give the AB stacking sequence of the graphite crystal structure. A stacking fault is created if the top sheet is shifted from this position, with the extreme case when all the atoms lie directly over the atoms in the lower graphene sheet, leading to a thermodynamically unstable AA stacking mode. Stacking faults of this type lead to asymmetric broadening and intensity changes in the XRD peaks of disordered materials.^29^ However, Johnsen *et al.* suggested that the complexity of the disorder in the graphite structure through staging transitions during lithiation is greater than that described by the stage models, as stacking faults cannot fully explain the observed changes in XRD peak widths.^30^ At the stage of complete lithiation, all galleries are filled with Li, and the graphene sheets are organized in a single configuration (AA stacking) with the six-membered carbon atom rings aligned directly on top of each other, with Li atoms positioned in a sandwich-type arrangement between six-membered C rings (Fig. 1b).

Although stage models, like the R–H and D–H models, provide an apparently reasonable explanation for the changes that are observed in XRD patterns collected during Li intercalation in graphite, in reality, Li intercalation into the graphite-based anode of a LIB is inhomogeneous, and several studies showed significant deviations from the crystallographic stage models.^8,23,31^ An accurate description of the structural changes during inhomogeneous Li intercalation is complicated by three main factors: (1) the turbostratic graphite stacking forms in the metastable state in addition to the AB stacking,^29,32–34^ as shown in Fig. 1a. The graphite layer occasionally forms random stacks as metastable structures because the rotated graphite layers stabilize interlayer interactions, reducing interlayer C⋯C repulsion. The formation energy difference for turbostratic stacking is only 1–3 meV/6C higher than the ground state of the AB stacking mode^33^; (2) Li insertion causes a local shift from AB to AA stacking. This causes local deformation of the planar graphene sheets around the inserted Li as the gallery height is increased (Fig. 1b).^35^ The C–C bond length in the six-carbon rings extends due to gain of an extra delocalized electron by coordinating Li.^36^ This local distortion exists in the D–H model but is not properly quantified; and (3) as schematically shown in Fig. 1c, the local structure can be affected not only by the Li atoms intercalated in a gallery but also by the Li arrangement in the neighboring galleries. When Li atoms are loosely packed, intercalated Li atoms separated by a certain distance (see “Site order and Li distribution” subsection below) can maintain the gallery's height across several empty sites, giving Li vacant regions which retain an LiC_6_-type layer spacing.^20^ In short, the diffusion barrier of a Li atom is affected by a large number of factors both in its immediate environment and elsewhere in the structure, so any model or simulation must take into account the movement of several Li atoms in several galleries, which enormously increases the required computing resources for most suitable types of atomistic simulation.

**Fig. 2:**
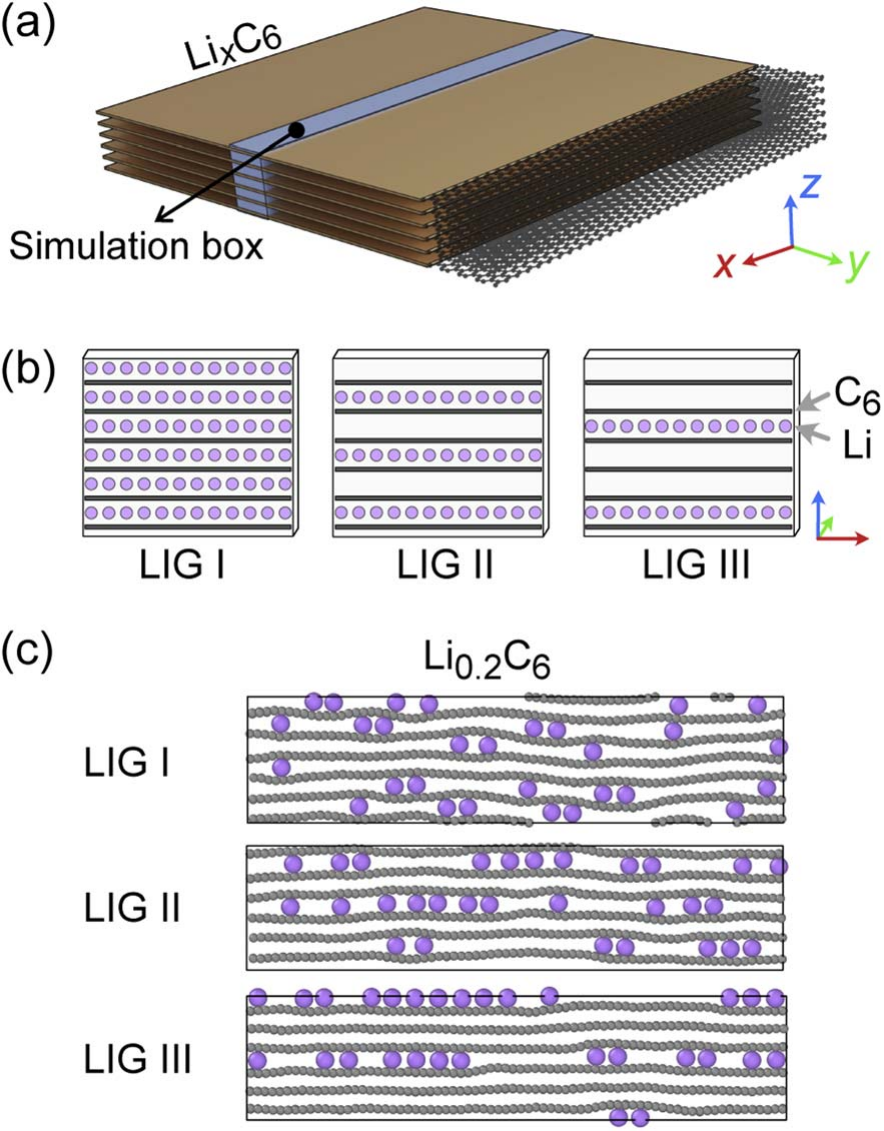
(a) Schematic representation of simulated Li_*x*_C_6_ stack. The simulation box is highlighted in light blue. In the simulation box, a single gallery can be filled with up to 24 Li atoms, thus, an LiC_6_ compound with a six-layer graphite structure would consist of 144 Li and 864 C atoms. The size was approximately 91.1 Å × 4.4 Å × 25.5 Å when *x* = 0, *P* = 1 atm, and *T* = 302.22 K. (b) Li-filled and empty galleries of LIG starting models I, II and III. (c) Snapshots of Li_*x*_C_6_ at *x* = 0.2 based on the three starting LIG models.

The sluggish kinetics of Li intercalation in graphite, due to the non-negligible covalent character of Li–C bonds,^37^ which make a Li atom sandwiched between two six-membered carbon rings highly stable, limit the usefulness of conventional MD. In addition, there are many possible diffusion pathways for any given Li atom in a gallery. Many degrees of freedom lead to very complex free energy landscape. Thus, it is challenging to efficiently sample the thermodynamic properties of such systems using MD. To overcome the time scale limitation of MD and sample a sufficient number of structural configurations to give reliable thermodynamic results, we performed REMD simulations. REMD (illustrated schematically in Fig. 1d and e.) involves running parallel, independent simulations on a set of non-interacting replicas of the same molecular system at various temperatures. Periodically, the calculation attempts to swap simulation models between different temperature regimes on the basis of Metropolis criteria,^38,39^ with acceptance probability:

1 *P* = exp[(*β*_*i*_ − *β*_*j*_)(*E*_*i*_ − *E*_*j*_)]

where *E*_*i*_ is the total potential energy of the replica *i* at temperature *T*_*i*_; *β*_*i*_ = 1/(*k*_B_*T*_*i*_) and *k*_B_ is the Boltzmann constant. This approach allows the acquisition of thermodynamic information at high temperatures to overcome free-energy barriers, which restrict Li-atom migration and graphite lattice shift at room temperature, while sampling at low temperatures remains accurate and unbiased. Moreover, prior knowledge of the Li diffusion mechanisms through a gallery is not required. We used REMD to expedite Li rearrangements and explore the free energy surface from systematically designed starting LIG models (see Fig. 2), setting a temperature range of 290.94 to 861.46 K. The results presented here are from the 302.22 K replica. The REMD simulations provided the structural configurations in the equilibrium state with a given Li concentration at a finite temperature.

## Computational studies of LIG

### Formation energy

Recent high-resolution microscopic studies demonstrated the dynamic mechanism of Li intercalation in graphite through non-equilibrium structural evolution.^22,40^ Furthermore, the layered structure of graphite leads to a Li concentration gradient in the in-plane direction, as Li flow occurs through a gallery.^31,41,42^ We rationalize that these experimentally observed structures are essentially snapshots resulting from the ensemble of local equilibrium states as the time scale of the Li rearrangement is substantially shorter than that of the Li rearrangement in the long-range order. In this study, we sought to identify the Li arrangements and energy landscape by comparing the locally stable states.

The basic structural model of a graphite stack is shown in Fig. 2a, with the simulation box used for REMD shown in blue. The periodic boundary condition of the simulation extends the model by repeating the box. Li migration through the graphene layers was not allowed as the results obtained by Langer *et al.*^43^ Additionally, no new C–C bond formation or breaking was observed due to the high temperature set and Li concentration.^44^ Li atoms were introduced systematically to create starting models corresponding to stages 1, 2 and 3 of the R–H model, denoted here as Li intercalated graphite (LIG) I, II, and III (Fig. 2b). LIG I has Li atoms spread through all the galleries, as might be expected in a fast dis(charge) rate.^8,26^ LIGs II and III contain Li-free galleries.

During the REMD simulations, we varied the Li concentrations in LIGs I, II, and III by randomly removing one Li atom from the model after 50 000 MD steps (see methods section for details) and relaxing to find the equilibrium Li distribution. This process was repeated until only carbon atoms were left—Fig. 2c shows representative snapshots of the partially lithiated composition Li_0.2_C_6_ starting from LIGs I–III at the relaxed stages.

**Fig. 3:**
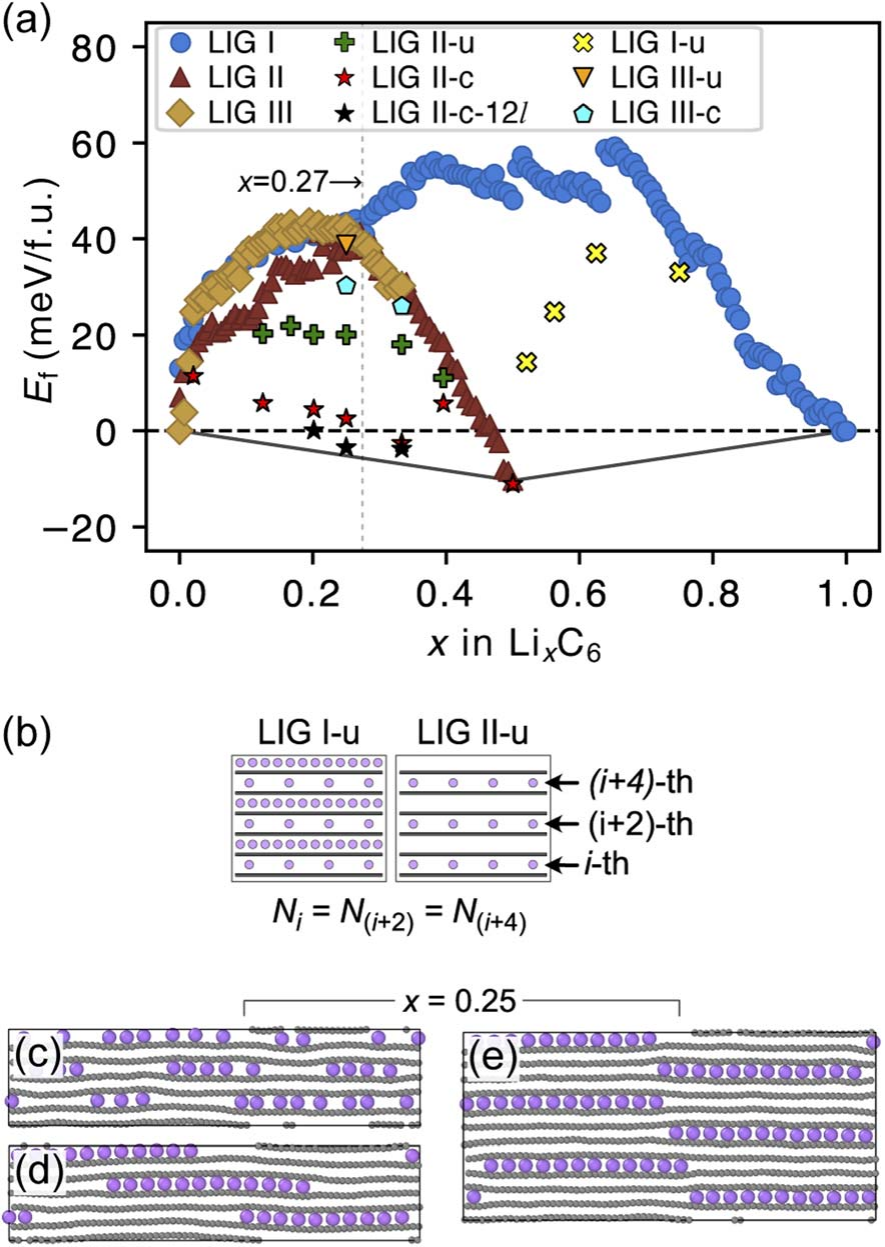
(a) Average formation energy as a function of *x* in Li_*x*_C_6_ at 302.22 K. Adding to LIGs I, II and III, LIG II-u marks the structural configuration where Li is uniformly intercalated through the filled layers, and LIG II-c marks *E*_f_ of the structural configuration in which all Li atoms in each plane are clustered. In addition, for *x* ≥ 0.5, LIG I-u marks the uniform Li intercalation in the partially filled galleries. The convex hull, including graphite, R–H stage 1, and R–H stage 2 compounds, is indicated by the black line (b) to model uniform Li intercalation through the Li-partially-filled galleries, we kept the same Li concentration at every second gallery. Representative snapshot of Li_0.25_C_6_ in (c) LIG II-u, and Li_0.25_C_6_ in (d) LIG II-c and (e) LIG II-c-12*l*. All Li atoms in each plane form clustering islands in 12-layer LIG II. Color code: light purple for Li and gray for C.

The formation energies of the LIG structures sampled from the REMD trajectories are presented in Fig. 3a. The formation energy *E*_f_ of Li_*x*_C_6_ compounds was calculated as

2 *E*_f_(Li_*x*_C_6_) = *E*(Li_*x*_C_6_) − (1 − *x*)*E*(C_6_) − *xE*(LiC_6_)

where the *E* values are the average energies of the sampled configuration, and *x* is the Li concentration in Li_*x*_C_6_. The equilibrium states of C_6_ (graphite), LiC_12_ of LIG II, and LiC_6_ of LIG I yield the construction of the convex hull for the Li_*x*_C_6_ formation energies. The observed trends of *E*_f_ values agree with previously reported density functional theory calculations.^14,15^

The *E*_f_ values for LIG I are similar to those for LIG II and III when *x* ≤ 0.27. This thermodynamic behavior suggests that the order of the vacant galleries is negligible for intermediates with low Li concentrations. The disordered Li vacancies induce internal strain in the Li-filled gallery, although *E*_f_ for the LIGs II and III benefit from van der Waals interactions in the empty galleries. *E*_f_ values of LIG I above the convex hull in all Li concentrations reflect phase instability. In particular, the highest difference above the convex hull for LIG I (*x* = 0.5) suggests that thermodynamically this configuration should undergo a phase transformation into LIG II, as LIG II is the most stable state at this Li concentration.

At *x* = 0.33 (corresponding to the stage 3 of the R–H model), the *E*_f_ value for LIG III is equivalent to that of LIG II, despite the fact that LIG III has a shorter average gallery height, suggesting stronger van der Waals interactions compared to LIG II (Fig. S1 in the ESI†). This result is attributed to the different lengths of covalent C–C bonds between the pristine (graphite-like) and Li-interacting (LiC_6_-like) carbon rings in graphene sheets. The *x*- and *y*-axis length of the LIG III simulation box increased more than those of LIG I and II with increasing in Li concentration (Fig. S2 in the ESI†) because every third graphite gallery is completely filled with Li and the direct interactions between Li and the six-membered carbon rings in the graphene layers elongate the C–C bonds. The pristine graphene layers between two empty galleries in LIG III have shorter C–C bonds and this is associated with an energy increase. It may also induce a stacking fault as intercalation with more Li atoms increases the count of slight ring offsets, which correspond to turbostratic stacking disorder (see subsection “Graphite stacking” and Fig. S3b in the ESI†).

We noticed that the *E*_f_ values of LIG II are slightly lower than those of LIG I and LIG III when *x* ≤ 0.27 (picked out with a vertical dotted line in Fig. 3a). Thus, we investigated the impact of a uniform distribution of Li in LIG II on *E*_f_. This configuration is denoted LIG II-u (Fig. 3b). We placed non-clustered Li in the partially filled galleries with the same concentration and performed the REMD simulations until the energy converged. Uniform filling of galleries with Li provides some additional stabilization. At *x* = 0.25 (Fig. 3c), the value of *E*_f_ decreased by about 20 meV f.u.^−1^ for LIG II-u compared to the corresponding value for LIG II. This reduction of *E*_f_ is also observed for the LIG I-u configuration at *x* > 0.5, which consists of full galleries alternating with partially filled galleries containing only isolated Li.

When we equilibrated at 302.22 K with an initial Li distribution based on LIG II with deliberately clustered Li atoms, we could obtain clustered “islands” (Fig. S4 in the ESI†). The clustered configurations are denoted LIC II-c and LIG II-c-12*l* (where 12*l* marks the inclusion of 12 graphene layers in the simulation box; in all other cases, six graphene layers were used). In LIG II-c (Fig. 3d), the 6 layers in the simulation box did not allow a clear pattern of offsets between the Li islands to build up during REMD simulations, but the 12-layer simulation, LIG II-c-12*l* (Fig. 3e) formed a clear offset pattern of Li islands similar to that described in the D–H model, resulting in a lower *E*_f_ value. These configurations at *x* = 0.25 gave respectively *E*_f_ values 18 and 24 meV f.u.^−1^ lower than the *E*_f_ of LIG II-u. These configurations also minimize the *y*-axis expansion of the simulation box. The islands of clustered Li form an offset pattern, even when they are separated by Li-free galleries. At a concentration of *x* = 0.4 the difference in *E*_f_ between LIGs II-u and II-c becomes small. This suggests that the Li-islands become so closely packed at this concentration that the graphene sheets in the Li-free regions of the galleries are pushed apart in the same way as for scattered Li configurations at lower concentrations (Fig. S5 in the ESI†). The result is a stage-2 type configuration which loses the van der Waals stabilization of the closed, graphite-like gallery regions and also has Li vacancies in gallery areas with LiC_6_-type spacing.

### Graphite stacking

During the lithiation process, the changes in local gallery height described above are accompanied by a change in stacking mode (also localized around the areas of Li intercalation) from AB in the graphite-type regions to AA stacking in the LiC_6_-type regions. We used this observation to investigate the correlation between the stacking mode and Li concentration by analyzing the relative positions of carbon atoms in neighboring graphite layers in the REMD sampled LIG structures.

**Fig. 4:**
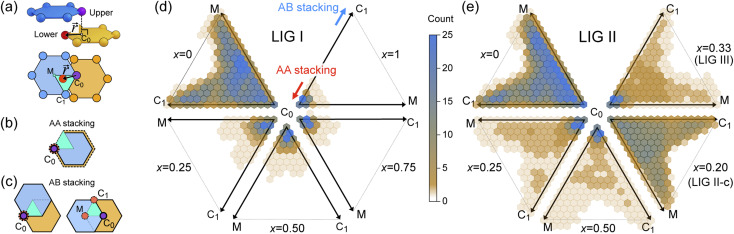
(a) Relative placement, 
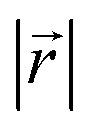
. The carbon atoms (red sphere) closest to C_0_ in (b) AA and (c) AB stacking. The cyan triangle denotes the observed projection plane in the upper ring. Observation distribution of 
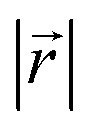
 of (d) LIG I and (e) LIG II/III of Li_*x*_C_6_.

To analyze the local stacking changes we consider the displacement of a carbon atom in a graphene layer relative to a six-membered carbon ring in the layer below (Fig. 4a). If we project a carbon atom from the upper layer (purple sphere) onto the lower layer, we can then measure the offset from the closest carbon atom (red sphere) in the lower six-membered carbon ring. The vector from the projected point (C_0_) to the carbon atoms is denoted 
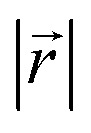
. A perfect AA stacking would consist entirely of 
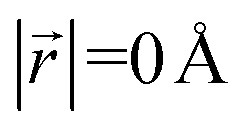
 (Fig. 4b), and AB stacking (the graphite crystal structure) would result in 
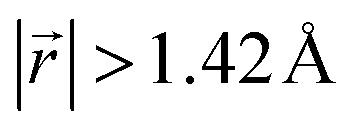
 for all ring shifts (Fig. 4c). The distortions in the LIG structures can lead to a range of 
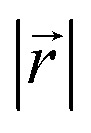
 values between 0 and 1.42 Å. All possible offset carbon positions were mapped onto a single equilateral triangle C_0_–C_1_-M in the six-membered carbon ring. This triangle is marked in cyan in Fig. 4a–c. The distribution of offset positions within the triangle for all rings in the simulation box at different values of *x* in Li_*x*_C_6_, for several different starting Li distributions, are shown in Fig. 4d and e.

At *x* = 0 in Li_*x*_C_6_ (Fig. 4d and e), we can see a wide distribution of ring offsets showing that the REMD model is capable of capturing the turbostratic stacking that exists in Li-free graphite.^29,32^ In the presence of Li atoms, regions of AA-stacking as found in the crystal structure of LiC_6_, immediately become apparent in the analysis. This structure is predominant in LIG I even at low Li concentrations (*x* ≤ 0.25), as shown in Fig. 4d. This observation means that even low concentrations of Li create large regions of AA-stacking. Inspection of MD snapshots shows that these result from opening sections of the galleries where there is a short distance between the Li-occupied sites (see subsection Site order and Li distribution). This increasing gallery height weakens the van der Waals interactions, and subsequently, prevents the stabilization of the empty regions. Ring offset analysis of LIGs II and III shows an increased preference for AB stacking (Fig. 4e), compared to LIG I. This is due to the count of 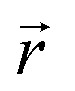 in the Li-free galleries, in which the AB stacking mode is favored. However, the general trend for the formation of AA stacking regions with increasing Li concentration is still clear.

This transformation of the stacking mode suggests that the shift of graphene layers is initiated locally at a low Li concentration, even in partially Li-filled galleries. Simultaneously, AB stacking would still be observed as long as there are continued (non-local) empty galleries as they are in the LIG II and III configurations, while the presence of scattered Li atoms in all galleries at low Li concentrations is sufficient to allow regions of AA stacking. Meanwhile, local turbostratic-like stacking or distortion 
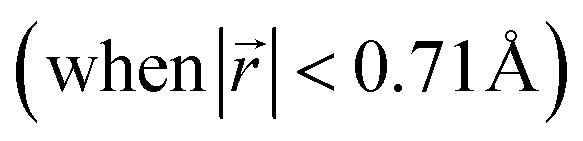
 helps to reduce the instability of the sites at which no Li is occupied but the adjacent Li-occupied site is associated with the AA stacking mode, as observed in LIG I. The intermediate region underwent thermodynamical relaxation by adapting the local structure distortions, mitigating the gallery height differences and stacking modes from a Li-occupied site to the adjacent Li-free site.

Ordered Li arrangement and uniform Li intercalation expanded the AB stacking mode. For example, when *x* = 0.25 in LIG II-u and LIG II-c-12*l*, the number of carbon atoms with 
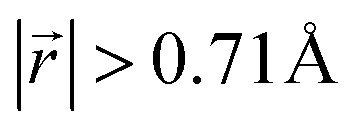
 increases to approximately 60% (Fig. S3 in the ESI†), and the formation energies are reduced in comparison to LIG II (*E*_f_ values for LIG II-u and LIG II-c-12*l* are represented by green crosses and black stars in Fig. 3a, compared to the red triangles for the *E*_f_ values of LIG II). The scattered distribution of Li in LIG II weakens the van der Waals interactions between graphene layers because the small and scattered Li clusters cause increased average gallery height at Li-free sites with Li on either side and curvature of the graphene layers (leading to an intermediate level of ring offset) in areas where the gallery height relaxes towards graphite-like values. For LIG II-c, by contrast, a lower number of carbon atoms with 
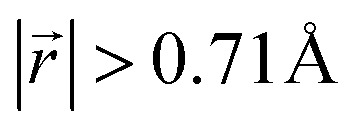
 was found compared to LIG II-u and LIG II-c-12*l*, although the Li islands are well clustered and the Li-free sites were continued. This AB stacking mode decrease indicates that the stacking mode transition is also associated with the offset pattern of Li clusters. An offset of Li clusters between next-neighboring galleries forms a curvature on either side layer, and the curved layer aids the stacking transition. However, the Li islands in LIG II-c did not form a clear offset pattern. Subsequently, the prolonged overlap of Li islands between next-neighboring galleries appeared and the flat Li-filled layers impeded the stacking mode transition in the adjacent Li-free site. Despite the decrease of AB stacking mode, the formation energies of LIG II-c are lower than LIG II-u (*E*_f_ values for LIG II-c are represented by red stars in Fig. 3a). This indicates the less significant impact of AA stacking mode on the formation energy when the gallery heights are relaxed. Therefore, a larger amount of the AB stacking mode is generally indicative of highly ordered Li clustering with locally ordered offset Li islands which allow the empty gallery regions to relax to a graphite-like height stabilized by van der Waals interaction.

### Site order and Li distribution

**Fig. 5:**
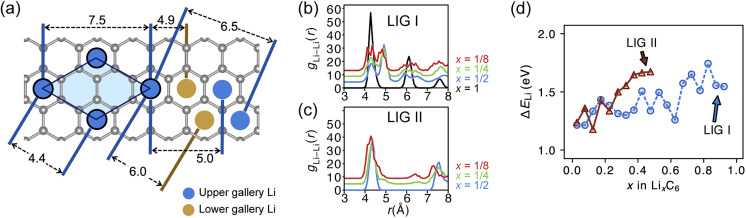
(a) Schematic of ordered Li arrangements formed in the LiC_6_-type configuration (blue circles in the light blue area) and distances between the selected Li atoms shown relative to single graphene sheet (grey hexagons). Blue circles represent Li above the sheet and brown circles below. Li–Li pair distribution function of Li_*x*_C_6_ in (b) LIG I and (c) LIG II. During the analysis, we accounted for the periodicity only in the *x*- and *z*-axes to avoid self-pairing of symmetry-generated Li atoms along the *y*-axis of the periodic simulation box. (d) Average binding energies of Li atoms in LIGs I and II. The Li binding energy in graphite was calculated as Δ*E*_Li_ = *E*_LIG+(*n*−1)Li_ + *μ*_Li_ − *E*_LIG+*n*Li_ where *E*_LIG+(*n*−1)Li_ and *E*_LIG+*n*Li_ are the average potential energies of the LIGs with (*n* − 1) and *n*Li atoms intercalated between graphene layers, and *μ*_Li_ is the average Li chemical potential calculated as the body-centered cubic crystal.

The REMD relaxation studies starting from any Li arrangement allowed us to analyze the ordering of Li-filled sites in the various Li distributions. When starting from LIGs I and II, the Li clusters, which are small compared to those that emerge from the pre-clustered starting point of LIG II-c, still have their first neighbor Li–Li interatomic distance at about 4.4 Å (Fig. 5a) in the plane of the galleries, as in the LiC_6_ crystal structure. The computed Li–Li partial pair distribution functions at different Li concentrations for LIGs I and II are shown in Fig. 5b and c, respectively. The Li partial pair distribution functions describe the local Li site order. Li–Li pairs at distances of 4.4, 6.0, and 7.5 Å appeared at high Li concentrations, and are attributed to Li islands with a well-ordered local Li arrangement. At low Li concentration, Li–Li pairs with distances of 4.9 and about 6.5 Å become noticeable. These pairs are more common in disordered Li arrangements. In particular, the pair at 4.9 Å in LIG I corresponds to two Li atoms separated by a graphene sheet shifted to sit over adjacent six-membered carbon rings in an offset arrangement (see Fig. 5a). This offset Li distribution avoids an overall increase in the *z*-axis of the simulation box (mechanical swelling). Furthermore, the pair distance around 5.0 and 6.5 Å is attributed to the first neighbor interatomic distance in the loosely packed clusters as the Li concentration decreases. As the Li concentration decreases, more sites are available for Li occupation. Subsequently, the Li atoms were dispersed unless the Li-free sites between a first neighbor were stabilized by closed, graphite-like galleries with AB stacking.

As the local environment changes with increasing Li concentration, the Li binding energy increases, as shown in Fig. 5d. The higher value of the binding energy (Δ*E*_Li_) indicates stronger Li–C interactions; therefore, higher energy is required to move the Li atom. In LIGs I and II, the lower values of binding energy at low Li concentrations reflect that the disorderly clustered Li induces structure deformation, consequently reducing the Li–C attractive interactions and increasing the total energies (less favorable); we speculate that the irregular increase is a result of the random removal of Li because the removal of Li was carried out without considering the probability of insertion/extraction processes between the structures at varying Li concentrations, whereas we can expect that the process of Li reconstruction pertains across the adjoining regions in a real graphite anode in which Li migration is a series of Li insertion/extraction. Therefore, Li migration will be hindered at high concentrations. The gap in the binding energy curves for LIG I and II is distinct when 0.27 < *x* < 0.50. The average Li binding energy in LIG II, which has empty galleries, is higher than that of LIG I, because empty galleries can reduce local strain and lead to less severe structural deformation than in LIG I. This comparison suggests that LiG I may transform into LiG II as Li atoms migrate during relaxation in expanded graphite layers, such as a real graphite anode. Meanwhile, an LIG II configuration would keep the Li-free galleries because Li atoms bind more strongly.

In general, reconstructive transitions (Li transport and Li island growth) are slower than displacive transitions (opening and closing of the galleries). The slow Li reconstruction kinetics may prevent a complete phase transformation^45^ unless significant relaxation time is allowed for the Li to fully rearrange. This is not the case during most charging and discharging of batteries. Our simulations suggest that the slow rate of reconstruction either leads to or is affected by kinetic trapping of Li due to distortions of the graphene sheets caused by Li arrangements in neighboring galleries. For example, during the REMD simulations, even though LIG II-c and LIG II-u were given the same number of Li atoms and arrangement of empty galleries, and LIG II-c is a more energetically stable structure, LIG II-u did not rearrange fully into clusters to resemble LIG II-c. This behavior occurs because forming a large Li cluster in one gallery in LIC II-u can cause the Li atoms in the neighboring galleries to be scattered during the resulting Li reorganization, breaking up any small clusters which may have formed and leading to a less stable configuration overall (Fig. 3c). Also the deformations of the graphite sheets around a large Li cluster push neighboring galleries shut, creating barriers to Li diffusion. Thus, although ordered arrangements like LIG II-c are energetically more favorable, their formation may be impeded in real systems by the dynamic factors of Li transport, varying gallery height and shifting stacking mode.

## Conclusions

We have used the REMD method to study the stability of Li intercalated in graphite by starting from different Li concentrations and distributions. REMD allowed us to explore conformational preferences with explicit consideration of atomistic structures at the given concentrations and distributions under both homogeneous and inhomogeneous conditions within a reasonable time period and at a relatively low computational cost. The models provide a good description of the three main features that control structural stability and Li transport: Li distribution, graphite stacking mode and gallery height. In contrast to earlier models we find these features to be very dynamic and also very dependent on one another (*e.g.* Li arrangements in neighboring galleries can cause or prevent the formation of Li clusters in a gallery) and it is not always possible to reach the lowest possible energy configuration for a certain Li concentration from any given starting distribution, even in very long simulations. By starting from clustered distributions of Li we have shown that these are energetically favorable and also that the clusters will cooperatively offset from one another to reach the most stable final combination of closed galleries with AB stacking and clusters with AA stacking, minimizing local distortions in the graphene sheets. This process leads to local arrangements that not only resemble the crystallographic stage models, but can also adapt to varying Li concentrations without the massive and organized long-range rearrangements required to change between stages in the R–H and D–H models. We believe this local ordering can provide an explanation of the phenomena observed in *operando* XRD data during stage transitions, as well as changes observed during open-circuit relaxation periods. It is important to note, however, that any kind of ordering takes time to form and the dynamic changes that occur during charge and discharge of a real battery work against this relaxation process. Our simulations indicate a fine balance between factors that stabilize the structures thermodynamically (closed galleries with AB stacking stabilized by van der Waals bonds, Li clusters, Li binding strongly between two six-membered carbon rings in an AA stacking sequence) and the competing requirement for Li to diffuse through the structure despite the obstacles of strong Li binding, stable Li clusters and graphene sheet distortions caused by the Li arrangements in neighboring galleries. We can also use the models to quantify all the relevant structural features in order to determine, interpret and understand stability for any Li concentration or starting arrangement. With a more realistic understanding of how Li intercalates into graphite, we hope that we and other researchers will in future be able to rationalize some of the currently unexplained behavior of graphite during battery cycling.

## Computational details

### Molecular dynamics simulation

MD simulations used the ReaxFF reactive force field in the isothermal-isobaric (NPT) ensemble, where the number of atoms (*N*), the pressure of the simulation box (*P*), and the target temperature (*T*) were kept constant. We adopted the set of reactive force field parameters which Raju *et al.* developed to describe Li interactions in carbon-based materials for the storage of electrical energy based on van der Waals corrected density functional theory.^46^ Their large-scale atomistic simulation using the force field provided the energetics and kinetics of Li intercalation in graphite consistent with the observations from the experiments for the MD simulations. The ReaxFF method employs a bond order/bond energy relationship, allowing the bond formation and dissociation during MD simulations.^47^ Interestingly, the ReaxFF MD simulations reproduced the increasing C–C bond length by the coordination with Li,^36^ benefiting from the update of the bond orders during the MD steps (Fig. S2 in the ESI†). Although the set of ReaxFF force field parameters reproduced the interlayer spacing of fully Li-filled LIG (LiC_6_) as DFT calculations (3.9 Å) when optimizing the structure, we observed an overestimation of the average interlayer distance of 4.3 Å at a temperature of 302.22 K due to thermal fluctuation in the simulations.

Each simulation used structures that consisted of graphene layers and Li atoms, and a single gallery filled with up to 24 Li atoms, in an orthorhombic simulation box with periodic boundary conditions in all directions. For example, a LiC_6_ compound with a six-layer graphite structure includes 144 Li and 864 C atoms. The anisotropic barostat allowed for anisotropic volume variation with varying Li concentrations. The integration time step was 0.5 fs and the Nose–Hoover temperature and pressure couplings were applied with damping constants of 100 and 1000 fs, respectively.

### Replica-exchange

We used 32 replicas in each REMD simulations, setting a temperature range of 290.94 to 861.46 K.^48^ The specific temperatures were 290.94, 302.22, 313.84, 325.81, 338.14, 350.84, 363.94, 377.45, 391.38, 405.73, 420.52, 435.77, 451.48, 467.68, 484.38, 501.58, 519.32, 537.62, 556.48, 575.91, 595.93, 616.58, 637.85, 659.75, 682.37, 705.68, 729.71, 754.48, 780.03, 806.34, 833.47 and 861.46 K. The highest temperature was set to avoid free Li movement in the graphite gallery by thermal energy (*k*_B_*T*/*E*_a_ = 0.11, where *k*_B_ is the Boltzmann constant, *T* is the temperature and *E*_a_ is the activation barrier for Li diffusion). Representative REMD simulations and the exchanges of their replicas in the temperature space are presented in Fig. S6 in the ESI.† MD simulations were carried out using the parallel reactive molecular dynamics module in the LAMMPS package.^49–52^ Before the REMD simulations, each fully Li-intercalated LIG system was equilibrated for a 40 ps NPT run at 1.0 atm and 302.22 K. The temperature exchange was attempted every 500 MD steps. We visualized the trajectories using Ovito.^53^ Regarding the convergence of the method, to see whether the thermodynamic quantities produced by the REMD simulations converge in the equilibrium distribution, we compared the average potential energies at 302.22 K of the MD simulation results by performing REMD simulations for the fixed-Li-concentration Li_*x*_C_6_ compounds until the average energy difference was less than 0.5 meV per atom over 200 000 MD steps. We conducted a series of simulations across the range of possible Li concentrations for LIGs I, II, and III by randomly removing one of the Li atoms from the model after every 50,000th REMD step until only C atoms remained. We equilibrated for 10 000 fs (20 000 REMD steps) after each Li-removal process to sample the structures. The representative comparison of potential energy evolution between regular MD and REMD is presented in Fig. S7 in the ESI.†

### Stacking analysis

Using the REMD trajectories, we analyzed the displacement of each carbon atom relative to the closest ring in the neighboring graphene layer. We sampled 64 structures at each available Li concentration. The neighbor-finding approach of the Pymatgen package^54^ was used to analyze local structures for individual carbon atoms in the lower graphene layer. This near-neighbor method is intended for periodic structures meaning that we could consider atoms in cells adjoining the main suimulation box.

## Data availability

The data and analysis code used in this work can be downloaded at https://doi.org/10.6084/m9.figshare.24948147. Due to the large size of the data, only selected data were uploaded.

## Author contributions

A. Y. K. conceived the study and supervised the project. H. P. performed the model building, REMD simulations, post-process calculations, data curation and visualization. H. P., D. S. W., and A. Y. K. contributed to the analysis, data interpretation, and manuscript writing.

## Conflicts of interest

The authors declare that they have no competing interests.

## Supplementary Material

SC-015-D3SC06107H-s001

SC-015-D3SC06107H-s002

SC-015-D3SC06107H-s003

SC-015-D3SC06107H-s004
