# Supplementary material for: Replica exchange molecular dynamics for Li-intercalation in graphite: a new solution for an old problem
Source: Chem Sci. 2024 Jan 16;15(8):2745–54. doi: 10.1039/d3sc06107h (PMC10882458; doi:10.1039/d3sc06107h)
Supplement: SC-015-D3SC06107H-s001 [file SC-015-D3SC06107H-s001.pdf]

Electronic Supplementary Material (ESI) for Chemical Science.
This journal is © The Royal Society of Chemistry 2024

Supporting Information: Replica Exchange Molecular Dynamics for Li-intercalation in Graphite: A new solution for an old problem

Heesoo Park,[*,†] David S. Wragg,[†,‡] and Alexey Y. Koposov[*,†,‡]

†*Centre for Material Science and Nanotechnology, Department of Chemistry, University of Oslo, 0371 Oslo, Norway*

‡*Department of Battery Technology, Institute for Energy Technology (IFE), 2027 Kjeller, Norway*

E-mail: heesoo.park@smn.uio.no; alexey.koposov@kjemi.uio.no

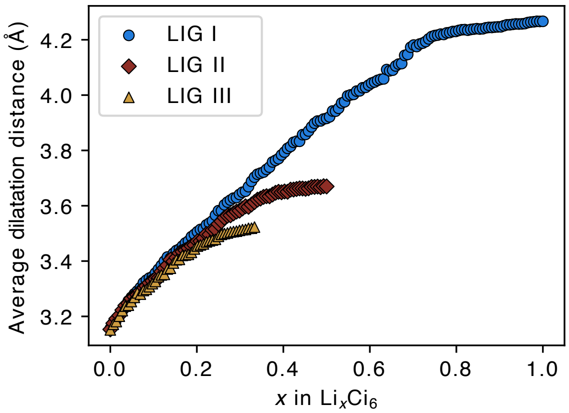

Fig. S1: Comparison of average gallery height of the graphite. (average height) = (size of the simulation box in the z-axis) / (number of graphene layers).

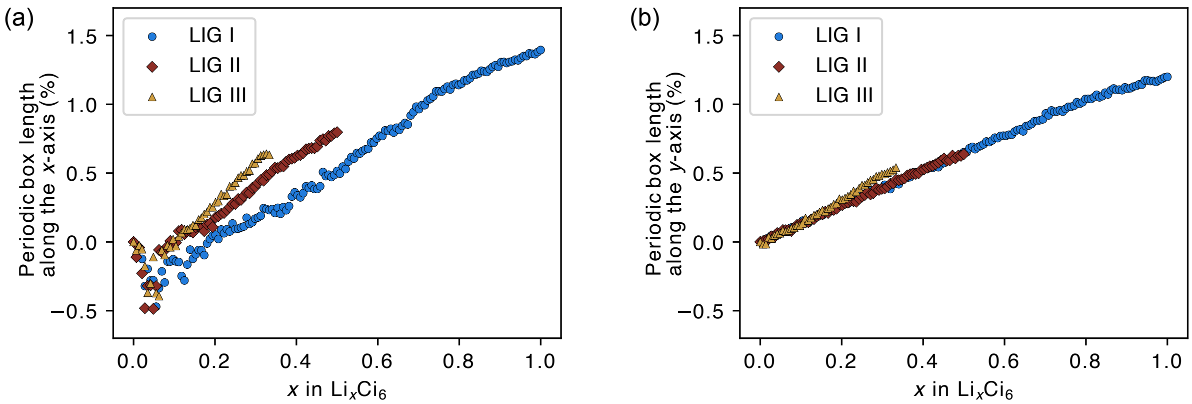

Fig. S2: Comparison of (a) x- and (b) y-axis length change of the simulation box of the sampled REMD simulations.

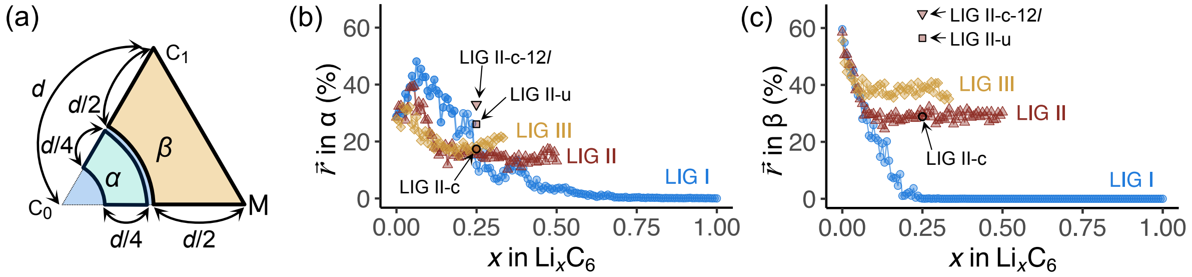

Fig. S3: Observations of $\vec{r}$ as a function of the Li concentration. To plot the $\vec{r}$ counts, we divide (a) the triangle C_0-C_1-M into three regions. The observations of the relative positions were classified as we define the subdivisions of α and β. Region α represents a small offset of between $d/4$ and $d/2$. Region β represents offsets between $d/2$ and d (where $|\vec{r}| = d$ in the direction $\mathbf{C_0M}$ or $\mathbf{C_0C_1}$ would represent AB stacking. The offsets of less than $d/4$ are classed as AA stacking. Graphite ($x = 0$) appears to include 30% carbon atoms in α and 60% carbon atoms in β, such that upper layer carbon atoms are placed in the AB and turbostratic stacking modes. For $x > 0$, turbostratic and AB stacking modes disappear as the empty galleries are filled with Li adapt AA stacking. In particular, increases in the pattern of the α region offset at $0.2 \leq x \leq 0.33$ may indicate the stacking fault induced by different optimal C–C bond lengths between the pristine and Li-intercating graphite layers.

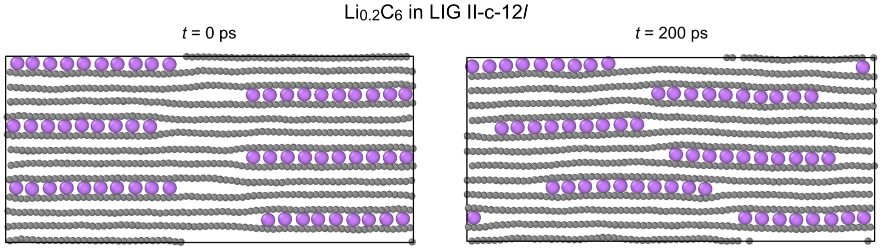

Fig. S4: Snapshots of $Li_{0.2}C_6$ in LIG II-c-12l. Color code: light purple for Li and gray for C.

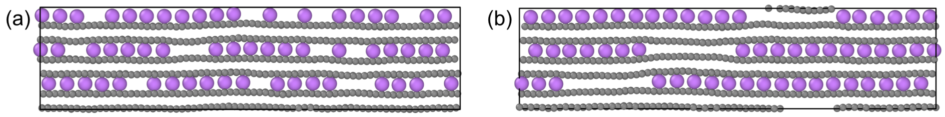

Fig. S5: Snapshots of $Li_{0.4}C_6$ in (a) LIG II-u and (b) LIG II-c. Color code: light purple for Li and gray for C.

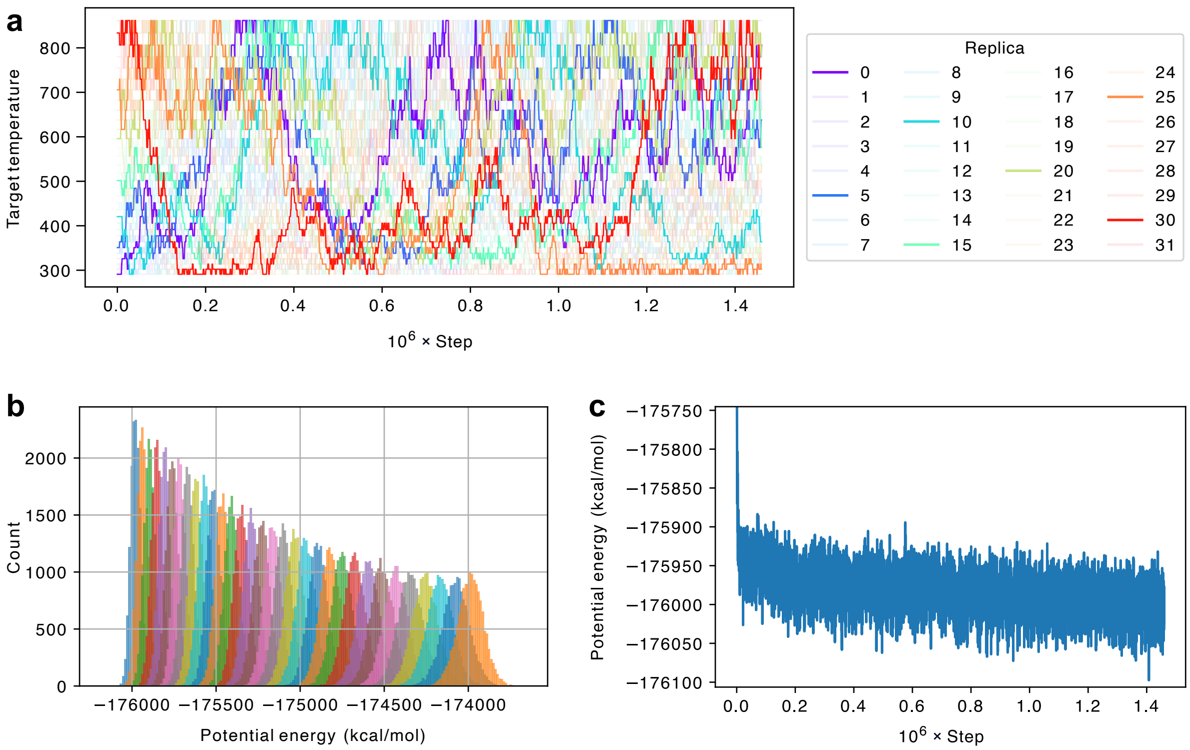

Fig. S6: Replica exchange molecular dynamics for $Li_{0.25}C_6$ (LIG II-u). (a) Exchanges of replicas in the temperature space. (b) Histograms of the total potential energies in an individual temperature space. (c) Minimum energy evolution during the REMD simulation.

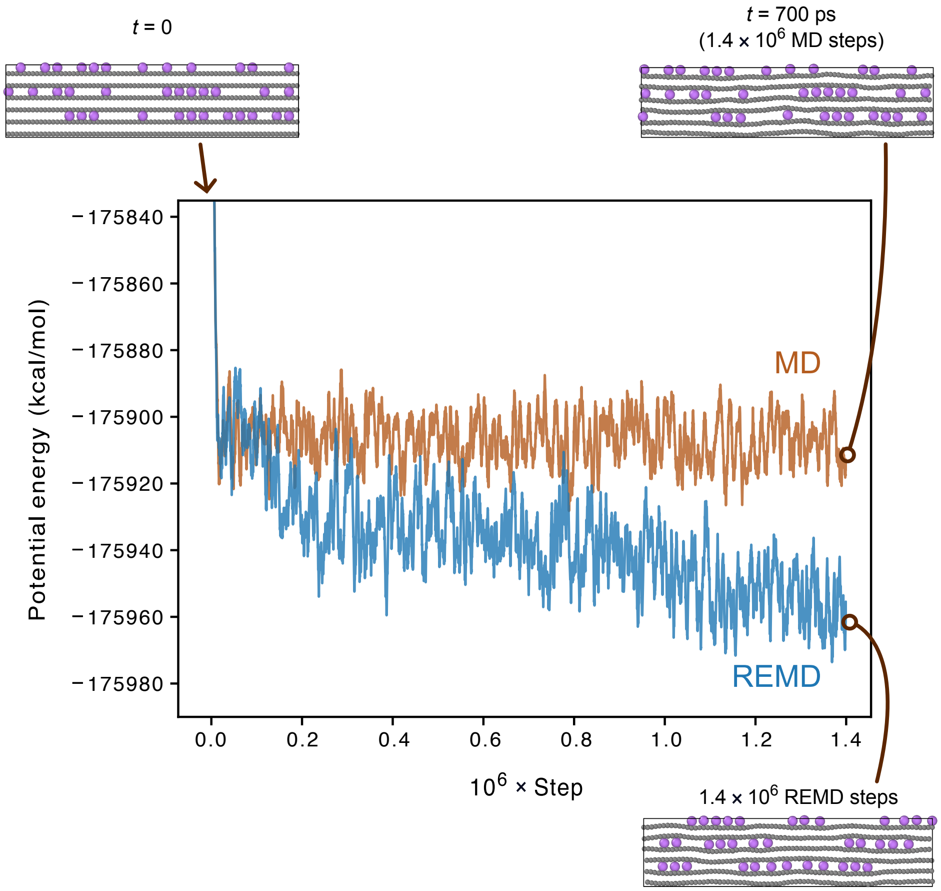

Fig. S7: Evolution of the potential energies in the MD (brown) and REMD (blue) simulations of LIG II-u at $x = 0.25$ and $T = 302.22$ K. The simulations were started with identical initial coordinates. The REMD simulation displays that Li clustering lowers the potential energies. The regular MD simulation, in contrast, shows no Li migration events in the given observation time, even though some relaxation of graphite gallery heights is observed.
